# Preface

**DOI:** 10.1093/jxb/erv037

**Published:** 2015-02-10

**Authors:** Nathalie González, Dirk Inzé

**Affiliations:** Ghent, Belgium

Size control of multicellular organisms such as plants poses a long-standing biological question that has fascinated scientists from every time and generation. Currently, the question on how size is measured and fixed during the growth of an organ or an organism is far from resolved, essentially because of its complex, integrated nature of regulation at the cellular, tissue, organ, and whole organism level.

Growth is a quantitative, dynamic, and multi-factorial trait regulated by numerous genetic and environmental factors. The study of this complex machinery therefore requires the integration of multiple approaches, or system biology approaches, at different scales (plant, organ, cell) including genetics, physiology, quantitative phenotyping, and various -omics technologies in order to obtain an holistic image of the molecular regulation of organ growth. In this special issue, recent approaches developed to unravel the regulation of plant organ growth and the knowledge obtained are presented for different parts of the plant (leaf, root, flower, fruit, and seeds).

The recent advances made in quantitative biology for the identification of the genetic basis of leaf growth regulation are first described and novel approaches that could be implemented for a better understanding of leaf growth and development are discussed (Gonzalez and Inzé). In the leaf, its final size is determined by cell proliferation and cell expansion that must be tightly co-ordinated. Hisanaga *et al.* integrated information from recent advances in molecular and genetic studies on compensation, an enhanced post-mitotic cell expansion associated with a decrease in cell number during lateral organ development is suggestive of such co-ordination.

Floral organs have been suggested, for a long time, to be leaf-like structures. As reviewed by Sablowski, links have emerged between floral homeotic genes and general regulators of lateral organ growth, such as the genes involved in organ patterning, tissue growth, and cell differentiation, which will help to understand the regulation of organ shape. Unravelling how fruit and seed sizes are regulated is important not only from a fundamental perspective but also from a more applied viewpoint. In the review from Azzi *et al*., current knowledge on the genes contributing to the regulation of various developmental processes governing fruit growth in tomato is presented. Li and Li have summarized current progress on the maternal control of seed size and discuss the roles of several newly identified regulators.

The study of how the growth of the underground part of the plant is regulated also represents an important challenge and new approaches have been developed to tackle this question as presented by Satbhai *et al.* who provide an overview of the major mechanisms, pathways, and genes that contribute to shaping root system architecture and its responses to environmental variation. Hormones play a major role as endogenous regulators of plant organ growth such as the roots. This is illustrated by the review from Pacifici *et al.* that summarizes recent findings on the molecular mechanisms on hormonal cross-talk and root meristem size and primary root growth control. In the review from Singh and Savaldi-Goldstein, particular attention is given to the brassinosteroid signalling pathway and its role in the regulation of root growth. Finally, Long *et al*. describe the SHR–SCR pathway which has a key role in regulating stem cell activities and radial patterning in the root, illustrating the importance of cell–cell communication in the regulation of growth processes.

We hope that this special issue on plant organ growth will further boost world-wide research on this highly interesting but challenging biological problem. Understanding size control is not only of academic interest but also opens numerous perspectives to improve crop yield thorough genetic modification, gene editing, and advance breeding.

